# Claudin-4 expression in gastric cancer cells enhances the invasion and is associated with the increased level of matrix metalloproteinase-2 and -9 expression

**DOI:** 10.3892/ol.2014.2295

**Published:** 2014-06-27

**Authors:** TSANN-LONG HWANG, TZU-TSUNG CHANGCHIEN, CHEE-CHAN WANG, CHI-MING WU

**Affiliations:** 1 Department of Surgery, Chang Gung Memorial Hospital, Tao-Yuan 33305, Taiwan, R.O.C.; 2 Department of Surgery, School of Medicine, Chang Gung University, Tao-Yuan 33302, Taiwan, R.O.C.; 3 Department of Cosmetic Science, Vanung University, Tao-Yuan 32061, Taiwan, R.O.C.

**Keywords:** claudin-4, gastric cancer, matrix metalloproteinase-2, matrix metalloproteinase-9, invasion

## Abstract

Claudin-4 is a member of a large family of transmembrane proteins known as claudins, which are essential for the formation and maintenance of tight junctions. Our previous studies have revealed that claudin-4 proteins are overexpressed in metastatic gastric cancer. To clarify the roles of claudin-4 in gastric cancer metastasis, human gastric adenocarcinoma (AGS) cells constitutively expressing wild-type claudin-4 were generated. Expression of claudin-4 in AGS cells was found to increase cell invasion and migration, as measured by Boyden invasion chamber assays. Moreover, the claudin-4-expressing AGS cells were found to have increased matrix metalloproteinase (MMP)-2 and -9 expression, indicating that claudin-mediated increased invasion may be mediated through the activation of the MMP protein. Overall, the results suggest that claudin-4 overexpression may promote gastric cancer metastasis through the increased invasion of gastric cancer cells.

## Introduction

Claudins are tight junctional proteins which are present at epithelial and endothelial cell membranes (1,2). Tight junctions form the primary barrier to the paracellular transport of solutes across the cells, and also play a critical role in establishing and maintaining epithelial cell polarity (3,4). Claudins are the major integral membrane proteins forming the backbone of tight junctions. The claudin family consists of 23 transmembrane proteins exhibiting distinct tissue- and development-specific distribution patterns (5).

Modulations in tight junction structure and function have been shown in epithelial tumorigenesis (6,7). A previous tissue microarray study showed that claudin-1, -3 and -4 are markedly expressed in the majority of intestinal-type gastric cancers, but are less frequently expressed in diffuse-type gastric cancer (8). Using cDNA microarray and immunohistochemistry analysis, our previous studies have shown that the expression of claudin-4 is significantly higher in intestinal-type than in diffuse-type gastric cancer (9,10). Other previous studies have also shown that claudin-2 expression gradually increases in the multistage process of gastric carcinogenesis (11,12). In addition, several other previous studies have reported aberrant claudin expression in various types of cancer. Specific examples include increased expression of claudin-3 and -4 in types of prostate and uterine cancer (13,14), high claudin-4 expression in pancreatic cancer (15), downregulation of claudin-7 in head and neck (16) and metastatic breast (17) cancer, and an increase in claudin-3 and -4 in breast cancer (18). However, the exact role of claudin overexpression and the functional importance of these proteins in the development of gastric cancer remain unclear.

Gastric cancer is one of the most common malignant tumors of the alimentary tract and is characterized by late clinical presentation, rapid progression and poor survival (19). The reason for this poor prognosis is that, at the time of diagnosis, gastric cancer usually shows extensive local tumor invasion and frequent spread to metastatic sites, particularly lymph nodes. Spread of malignant tumors is a multistep process and numerous stages of tumor invasion require degradation or breakdown of the extracellular matrix and connective tissue surrounding tumor cells (20,21). The matrix metalloproteinases (MMPs) are a family of zinc-containing enzymes which are involved in the degradation of various components of the extracellular matrix. In addition, there is considerable evidence to indicate that individual MMPs have important roles in tumor invasion and spread (22–27). Previous specific studies have suggested a major role for MMP-2 and -9 in the digestion of basement membrane type IV collagen, as an important mechanism for vessel invasion and metastasis in gastric cancer (28,29).

Previous studies have indicated modulatory effects of claudins on MMP activation. Agarwal *et al* showed that claudin-3 and -4 expression in ovarian epithelial cells enhanced invasion and was associated with increased MMP-2 activity (30). Oku *et al* showed that claudin-1 enhanced the invasive activity of oral squamous cell carcinoma cells by promoting cleavage of the laminin-5 γ2 chain via MMP-2 and membrane-type MMP-1 (31). Takehara *et al* demonstrated that the overexpression of claudin-4 specifically stimulated the invasive activity of colonic cancer cells and increased MMP-2 and -9 activity (32). Yoon *et al* found that claudin-1 is necessary and sufficient to induce cellular invasion in human hepatocellular carcinoma. In addition, the authors showed that activation of the C-Abl-PKCδ signaling pathway is critical for the expression and activation of MMP-2 and the subsequent induction of cellular invasion in response to claudin-1 expression (33). Using immunohistochemical analysis, the present study showed that the expression of claudin-4 was found to correlate with tumor invasion and MMP-2 and -9 expression in gastric cancer (34). AGS cells constitutively expressing wild-type claudin-4 were generated, and their effects on cell invasion and migration were studied. In addition, overexpression of claudin-4 in gastric cancer cells was shown to lead to increased expression of MMP-2 and -9, thus, suggesting a mechanism for the increased invasive potential of claudin-4-expressing gastric cancer cells.

## Materials and methods

### Cell culture and overexpression of claudin-4

AGS cells were purchased from the Food Industry Research and Development Institute (Hsinchu, Taiwan) and cultured in Ham’s F-12 medium containing 10% fetal bovine serum. Cells (2×10^5^ per well in a 24-well plate) were cultured for 24 h and used in the experiments. A full-length human claudin-4 cDNA was PCR-amplified from the cDNA of AGS cells and cloned into pcDNA3.1(+) (Invitrogen Life Technologies, Carlsbad, CA, USA). Transfection of AGS cells with plasmids was performed using Lipofectamine 2000 (Invitrogen Life Technologies), according to the manufacturer’s instructions. The stable transfectants expressing claudin-4 were selected by G418 (Sigma-Aldrich, St. Louis, MO, USA) and confirmed by immunoblotting analysis. Positive clones were maintained in the presence of 300 μg/ml G418.

### Expression construct

Full-length cDNAs for claudin-4 were PCR-amplified from AGS cells following RNA isolation and reverse transcription. For amplification of claudin-4, the following primers were used: Forward, CGGGATCCCTGA CAATGGCCTCCATGGGGCT; and reverse, GCTCTAGAT TACACGTAGTTGCTGGCAGC (35). The resulting PCR fragments of claudin-4 were cloned into *Bam*HI and *Xba*I restriction sites of the expression vector pcDNA3.1(+) (Invitrogen Life Technologies), and the sequence of all the constructs was verified by sequencing. The restriction enzyme sites recognized on the primers by *Bam*HI and *Xba*1 were forward, CGGGATCCCTGACAATGGCCTCCATGG GGCT and reverse, GCTCTA GATTACACGTAGTTGCTG GCAGC, respectively.

### Immunoblotting and densitometry

Confluent cell cultures were washed with Hank’s balanced salt solution (Invitrogen Life Technologies) and whole cell lysates were produced using lysis buffer: 62.5 mM Tris-HCl (pH 6.8), 10% glycerol and 2% SDS. Protein concentration was determined using the bicinchoninic acid kit (Pierce Biotechnology, Inc., Rockford, IL, USA). In total, 20 μg total proteins were separated by 10–20% SDS-PAGE on Tris-glycine gels (Invitrogen Life Sciences) and transferred to polyvinylidene difluoride membranes (Millipore, Bedford, MA, USA). The membranes were blocked with 5% non-fat dry milk, washed in Tris buffered saline and Tween-20 buffer (Pierce Biotechnology, Inc.) and probed with the primary antibody at the following dilutions: Rabbit polyclonal anti-human claudin-4, 1:500 (Abcam, Cambridge, UK); rabbit polyclonal anti-human MMP-2, 1:1,000; and rabbit polyclonal anti-human MMP-9, 1:1,000 (both purchased from Cell Signaling Technology, Inc., Beverly, MA, USA). The blots were then washed and incubated in horseradish peroxidase-conjugated secondary antibody (rabbit polyclonal anti-mouse IgG; 1:10,000; Amersham Pharmacia Biotech, Piscataway, NJ, USA). Enhanced chemiluminescence was performed using the enhanced chemiluminescence kit (Amersham Pharmacia Biotech) for detection. Scanning densitometry was performed using the Kodak 1D 3.6 program (Eastman Kodak, Rochester, NY, USA).

### Invasion and cell migration assay

The cell invasion capabilities of the claudin-4-overexpressing clone and AGS cells were determined using a modified Boyden chamber invasion (36). The ECMatrix™ insert (Chemicon, Temecula, CA, USA) of 8-μm pore size was coated with 25 μg/filter of Matrigel basement membrane matrix extracted from Engelbreth-Holm-Swarm mouse tumor (Chemicon, Temecula, CA, USA). Cells were cultured to ~80% confluency and serum-starved overnight. On the first day of the invasion experiment, cells were trypsinized and a viable cell count was obtained. In total, 3×10^5^ cells were plated into the top of each of the coated filters in serum-free medium. An equal volume of the same medium containing 10% FBS was placed in the lower chamber (the well beneath the filter) to act as a chemoattractant. The assay plate was incubated at 37°C for 48 h. Following incubation, the filters were fixed with 3% glutaraldehyde in phosphate-buffered saline and stained with crystal violet. Cells on the upper surface of the filter were gently scraped off, and those that had penetrated through the Matrigel to the lower surface of the filter were counted using a microscope (Olympus CX21LED; Olympus Corporation, Tokyo, Japan). Three independent experiments were performed with triplicate measurements. For assessing cell migration, the assay was performed essentially as described above, with the exception that the cells were plated on top of uncoated ECMatrix inserts.

### Statistical analysis

Data are presented as the mean ± standard error, calculated from at least three repeated groups in all experiments. The differences between groups were assessed by Student’s t-test, and P<0.05 was considered to indicate a statistically significant difference.

## Results

### Claudin-4 expression in AGS cells

**Figure 1:**
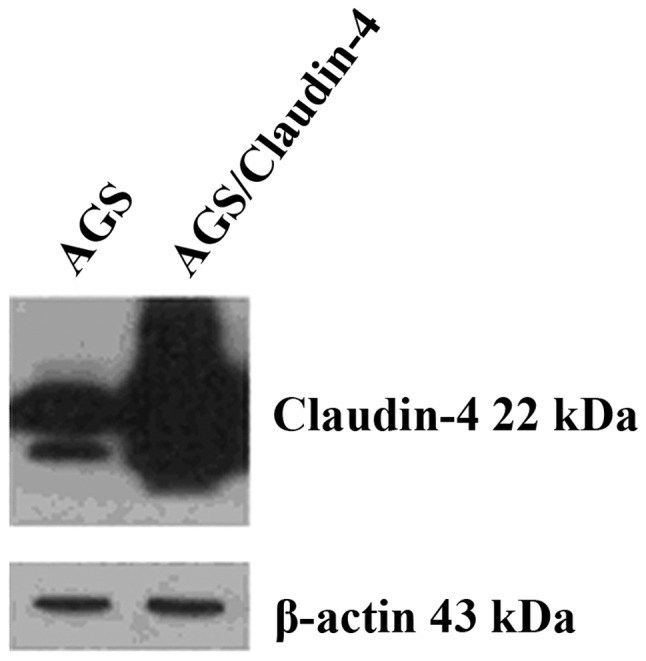
Immunoblot analysis of claudin-4 expression in control AGS cells and AGS cells stably transfected with full-length, wild-type claudin-4 (AGS/Claudin-4), conducted using anti-claudin-4 antibody. β-actin was used as an internal control.

To study the roles of claudin-4 protein overexpression in gastric cancer cells, the human adenocarcinoma cell line, AGS, was transfected with expression vector pcDNA3.1(+) encoding wild-type claudin-4 cDNA. Immunoblot analysis of the selected stable clones using claudin-4 specific antibody showed that the clone, AGS/claudin-4, expressed high levels of claudin-4 (Fig. 1).

### Claudin-4 expression enhances invasion and cell migration of AGS cells

**Figure 2:**
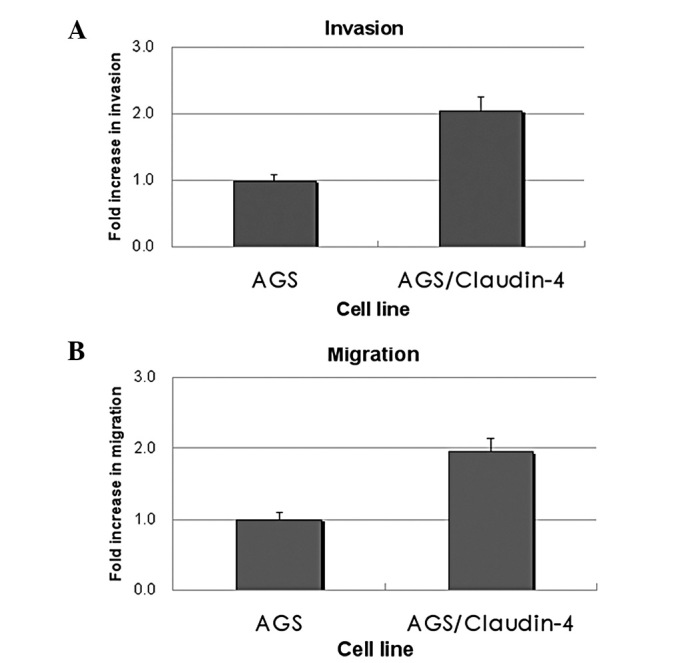
Effects of claudin-4 expression on the cell invasion and migration of gastric cancer cells. Modified Boyden chamber invasion assay was performed on AGS stable transfectants. The number of invading and migrating cells was measured after 24 h. The graphs present the mean number of (A) invading and (B) migrating claudin-4-overexpressing AGS cells (AGS/Claudin-4) compared with the control cells (AGS), as measured by cell counting.

Differences in invasion between claudin-4-expressing cells and control AGS cells were evaluated using a modified Boyden chamber invasion assay. Claudin-labeled cells were placed on Matrigel-coated ECMatrix inserts and the cells invading through Matrigel were analyzed after 48 h. The results showed that claudin-4-overexpressing AGS cells were significantly more invasive (2-fold increase) than the control AGS cells (P<0.05; Fig. 2A). In addition, the rate of cell migration between these two cells was also compared using the two-chamber assay with an uncoated insert. AGS cells overexpressing claudin-4 showed increased migration (2-fold increase) compared with the control AGS cells (P<0.05; Fig. 2B).

### Effects of claudin-4 overexpression on MMP-2 and -9 expression

**Figure 3:**
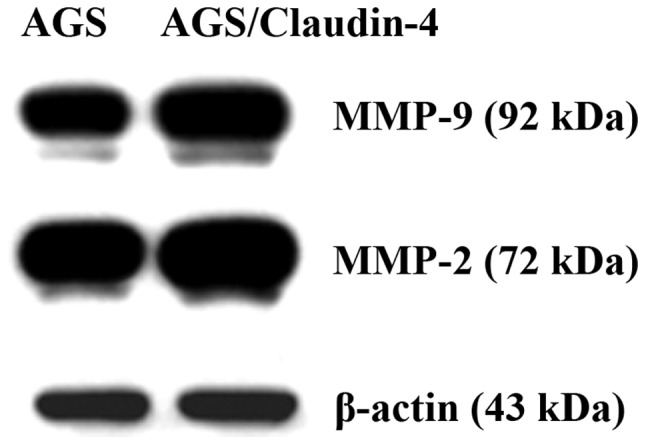
Immunoblot analysis of matrix metalloproteinase (MMP)-2 and -9 expression in stably transfected AGS cells using anti-MMP-2 or -9 antibodies. AGS cells stably transfected with full-length, wild-type claudin-4 (AGS/Claudin) expressed higher levels of MMP-2 and -9 compared with those in the control cells (AGS).

A previous study indicated the modulatory effects of claudins on MMP-2 activation in ovarian cancer cells (30). To understand the underlying mechanism of claudin-induced increased invasion of AGS cells, changes in MMP-2 and -9 expression were examined by immunoblotting analysis. AGS cells stably transfected with full-length wild-type claudin-4, expressed higher levels of MMP-2 and -9 than the control AGS cells (Fig. 3).

## Discussion

In our previous study, immunohistochemistry was used to examine the expression levels of claudin-4 and MMP-2 and -9 in 189 gastric cancer samples, and their correlation with tumor invasion and clinicopathological parameters was analyzed (34). It was found that the expression of claudin-4 was significantly higher in gastric cancer cases with advanced depth of wall invasion, lymph node metastasis, lymphatic invasion and high tumor node metastasis stage. Further analysis revealed that claudin-4 expression was found to significantly correlate with the expression of MMP-2 and -9. In the current study, human gastric adenocarcinoma cells, AGS, constitutively expressing claudin-4 were generated. Overexpression of claudin-4 in AGS cells was found to increase cell invasion and migration. Moreover, the claudin-4-expressing AGS cells were found to have increased MMP-2 and -9 expression. The results indicated that claudin-mediated invasion may be mediated through the activation of the MMP protein.

A similar effect has been observed in other types of cancer cells (30,32). Previously, Agarwal *et al* showed that claudin-3 and -4 expression in ovarian epithelial cells enhanced invasion and was associated with increased MMP-2 activity (30). In addition, Takehara *et al* demonstrated that overexpression of claudin-4 specifically stimulated the invasive activity of colonic cancer cells and increased MMP-2 and -9 activity (32). It is known that claudins affect cell physiology through recruiting signal transduction-related molecules at tight junctions (37). The carboxylic terminal region of claudin proteins contain a PDZ domain-binding motif that potentially interacts with a number of PDZ domain-containing proteins, such as ZO proteins (38,39). These interactions also serve as adapters for other proteins involved in cell signaling. A number of other cytosolic and nuclear proteins, including regulatory proteins (e.g. Rab3b), protein kinases (e.g. c-Abl-PKCδ) and transcription factors (e.g. ZONAB), have also been shown to interact directly or indirectly with tight junction complexes (33,40,41).

To further confirm the role of claudin overexpression on invasive properties of ovarian cancer cells, Agarwal *et al* previously performed siRNA-mediated knockdown of claudin-3 and -4 expression in the ovarian cancer cell line, OVCAR-5. Inhibition of claudin-3 and -4 expression in OVCAR-5 cells significantly reduced the invasive potential of these cells (30). However, siRNA-mediated knockdown of claudin-3 and -4 in OVCAR-5 cells did not lead to a decrease in the large amount of MMP-2 activity present in the cells. These results implied that the malignant ovarian cells may have additional or alternative pathways to active MMP-2 activity (30). Previously, Surgucheva *et al* and Nothnick investigated the importance of microRNA in MMP-9 regulation (42,43). Surgucheva *et al* identified targets for microRNAs in the 3′-untranslated region of MMP-9 involved in the regulation of MMP-9 expression (42). The authors then isolated microRNAs from the optic nerve A7 astrocytes and 293T cells and confirmed the role of mi340 in regulation using specific inhibitors and mimics. The results obtained showed a novel microRNA-mediated mechanism of MMP-9 expression regulation.

However, the opposite effect has been previously observed in pancreatic cancer cells (44). Michl *et al* showed that claudin-4 was overexpressed in pancreatic cancer and associated with decreased invasiveness *in vitro* and *in vivo*. In the authors’ ultrastructal studies, an increase in tight junctions was found between neighboring claudin-4-overexpressing tumor cells. This led to the conclusion that an increase in the density of cell-cell adhesions formed by tight junctions may present a crucial impediment against the dissociation of pancreatic cancer cells from the original tumor. This, in turn, is likely to prevent the invasion into neighboring tissues or formation of distant metastasis. A similar mechanism has previously been proposed for the E-cadherin-mediated development of epithelial polarity and suppression of the invasiveness of cancer cells, which has been associated with increased cell contact formation. Thus, an alteration in claudin-4 expression appears to play a role in the invasiveness of cancer cells, by modulating the barrier function of tight junctions or by mediating MMP-2 and -9 activity. However, the overall correlation between claudin-4 overexpression and the invasive capacity have not been fully elucidated. Additional studies have been warranted to investigate the correlation between claudin-4 overexpression and cell invasion in various cancer cells.

Previously, Surgucheva *et al* showed that an additional protein, γ-synuclein, also upregulated MMP-2 and -9 in retinoblastoma cells (45). γ-synuclein is a member of a family of small soluble proteins, which is involved in tumorigenesis since it is overexpressed in advanced infiltrating carcinomas of the breast (46). Notably, γ-synuclein stimulates metastasis, being a key positive regulator for cancer invasion and metastasis and a marker for malignant progression (47). The present study showed that claudin-4 is overexpressed in human gastric cancer cells and associated with increased cell invasiveness. Furthermore, the claudin-4-expressing gastric cancer cells were found to increase MMP-2 and MMP-9 expression, indicating that claudin-mediated increased cell invasion may be the result of MMP protein activation.
